# Optimising non-invasive screening for hepatic fibrosis in people living with HIV and intermediate FIB-4 scores

**DOI:** 10.3389/frhs.2026.1807690

**Published:** 2026-04-29

**Authors:** Luis Ramos-Ruperto, Maria Luisa Montes, Carmen Busca-Arenzana, Petru Manescu, Richard Gilson, Delmiro Fernandez-Reyes, Alejandro Arenas-Pinto

**Affiliations:** 1 HIV Unit, Internal Medicine Department, Unidad de VIH, Departamento de Medicina Interna, Hospital Universitario La Paz-Carlos III, IdiPaz, Madrid, Spain; 2 Centro de Investigación Biomédica en Red de Enfermedades Infecciosas (CIBERINFEC), Instituto Salud Carlos III, Madrid, Spain; 3 Centre for Clinical Research in Infection and Sexual Health, Institute for Global Health, University College London, London, United Kingdom; 4 Department of Computer Science, Faculty of Engineering Sciences, University College London, London, United Kingdom; 5 The Mortimer Market Centre, Central and North West London NHS Foundation Trust, London, United Kingdom

**Keywords:** elastography, fibroscan, HIV, liver fibrosis, machine learning, MASLD, screening, steatosis

## Abstract

**Background:**

Steatotic liver disease is increasingly recognised in people living with HIV. Non-invasive fibrosis screening strategies commonly rely on the FIB-4 score, but individuals with intermediate values (1.3–2.67) fall into a diagnostic grey zone where additional risk stratification may be required. Identifying simple and effective approaches to guide further assessment is particularly important in routine HIV care settings.

**Methods:**

We analysed two independent cohorts of people living with HIV undergoing transient elastography (TE): a development cohort from London (*n* = 229) and an external validation cohort from Madrid (*n* = 188). Among individuals with intermediate FIB-4 scores, we evaluated the performance of established non-invasive scores, including APRI, and several machine learning models for predicting liver stiffness thresholds associated with significant fibrosis (≥7 kPa and ≥8 kPa). Model performance was assessed using sensitivity, specificity, and predictive values.

**Results:**

In both cohorts, the prevalence of significant fibrosis was low. APRI demonstrated consistently high sensitivity and strong negative predictive value for identifying individuals without significant fibrosis. Machine learning models showed modest discrimination and tended to favour negative predictions, reflecting the low prevalence of fibrosis in this screening population. Across models, no machine learning approach demonstrated clear improvement over APRI in identifying individuals at risk of fibrosis.

**Conclusion:**

In people living with HIV with intermediate FIB-4 scores, APRI may provide a simple and effective strategy to further stratify fibrosis risk in routine clinical practice. In this real-world screening population, machine learning models did not outperform established non-invasive scores.

## Introduction

Because of the widespread use of effective antiretroviral therapy and the successful elimination of hepatitis C virus coinfection in many settings, Metabolic Dysfunction-Associated Steatotic Liver Disease (MASLD) has emerged as the leading cause of liver fibrosis among people living with HIV (1–3). While liver disease in this population was historically attributed to hepatotropic viral infections, alcohol and drug toxicity, the role of metabolic dysfunction is now better recognised. Moreover, people living with HIV are subject to additional mechanisms—such as oxidative stress, mitochondrial damage, microbial translocation, and subclinical inflammation—that may further contribute to the development and progression of liver disease (1).

Recent data highlight the growing burden of MASLD and liver fibrosis among people living with HIV. Recent systematic reviews and meta-analyses estimated that between 34% and 38% of people living with HIV had MASLD and 25% of those with MASLD presented with some degree of liver fibrosis (4, 5). Region-specific estimates reveal even higher rates, with a MASLD prevalence of 49% in both Spain (95% CI: 31%–66%) and the United Kingdom (95% CI: 7%–92%) (6). Steatotic liver disease (SLD) is also the leading cause of transaminase elevation in individuals following HCV cure (7).

MASLD is a progressive condition that can lead to fibrosis and cirrhosis. Clinical guidelines recommend non-invasive screening with serum-based fibrosis scores in patients with elevated liver enzymes (8, 9). Among these, the FIB-4 score is widely used due to its simplicity and accessibility, incorporating age, aspartate and alanine aminotransferases (AST and ALT), and platelet count (10). A score below 1.3 generally rules out significant fibrosis, while a score above 2.67 indicates the need for referral and further evaluation. However, patients with FIB-4 scores between 1.3 and 2.67 represent a zone of diagnostic uncertainty, where decision-making is less clear. Additional assessment, typically with transient elastography (TE), is recommended. Hepatic fibrosis, assessed by TE, has been linked with increased cardiovascular risk in people living with HIV, even in the early stages of liver damage (11), as well as its established association with hepatocellular carcinoma (HCC) (12).

People living with HIV represent a group at particular risk of liver fibrosis, and the diagnostic uncertainty associated with intermediate FIB-4 scores applies also to this population Addressing this gap is particularly important in routine HIV care settings, where fibrosis assessment often takes place outside specialised hepatology clinics and simple, accessible tools are needed to guide decisions on further investigation. In this study, we evaluated whether risk stratification could be improved by applying additional approaches beyond FIB-4, including both routinely available scores such as APRI and exploratory machine learning models. Our aim was to identify strategies that could optimise non-invasive screening, improve efficiency in referral pathways, and ultimately support earlier detection of liver disease in this high-risk population.

## Methods

The aim of this study was to develop machine learning models to predict liver fibrosis in adults living with HIV with intermediate FIB-4 scores defined as ranging between 1.3 (low probability of fibrosis) and 2.67 (high probability of fibrosis). An additional non-invasive marker, the APRI score, was included as a possible second stage after FIB-4 calculation.

### Hepatic fibrosis definition

Transient elastography (TE) was used as the reference standard for fibrosis assessment. TE was performed under fasting conditions using a FibroScan® device (Probe M, FS402; Echosens, Paris, France, http://www.echosens.com), with concurrent measurement of the controlled attenuation parameter (CAP) as a measure of steatosis.

All examinations were conducted by experienced operators following the manufacturer's protocol. TE results were expressed in kilopascals (kPa) as the median value of ten measurements. Measurements were considered unreliable if the interquartile range (IQR) exceeded 30% of the median value. To estimate fibrosis stages, we applied the following thresholds, consistent with previous protocols: 7.1 kPa for stage F2, 8.7 kPa for stage F3, and 10.3 kPa for stage F4 (13). Two thresholds were applied to define significant fibrosis: ≥7 kPa, based on previous studies (13, 14), and ≥8 kPa, as recommended by European clinical guidelines (8, 9).

### Non-invasive scores

FIB-4 scores >1.3 and <2.67 were considered to represent the diagnostic grey zone of this non-invasive marker for the detection of liver fibrosis. These cut-offs were selected based on clinical guidelines for the assessment of fibrosis in the context of SLD. For APRI score a value greater than 0.5 was used (8, 9).

### Study population and ethical approval

Two retrospective cohorts were used for model development and validation: a cohort from the Mortimer Market Centre, Central and North West London NHS Foundation Trust (London), with data collected between 2017 and 2023, and a cohort from Hospital Universitario La Paz (Madrid), which served as the external validation cohort using data collected between January 2017 and June 2018.

Ethical approval was obtained from the relevant institutional review boards for both cohorts. In the UK, approval was granted by the Health Research Authority (Ref 25/HRA/4006), and individual consent was not required in accordance with national regulations for retrospective studies. In Spain, the study was approved by the local ethics committee (code PI-2248, 03-2016), and all participants provided informed consent.

### Inclusion criteria

Participants were eligible for inclusion if they were over 18 years of age, had a confirmed HIV infection without hepatitis B virus coinfection, had at least one liver stiffness measurement obtained using TE, and had a FIB-4 and APRI score available at the time of the TE assessment. Most participants had persistent transaminase elevation for more than 6 months. Individuals with active hepatitis C virus coinfection were excluded, although a history of past HCV infection was permitted.

### Data preprocessing

**Figure 1:**
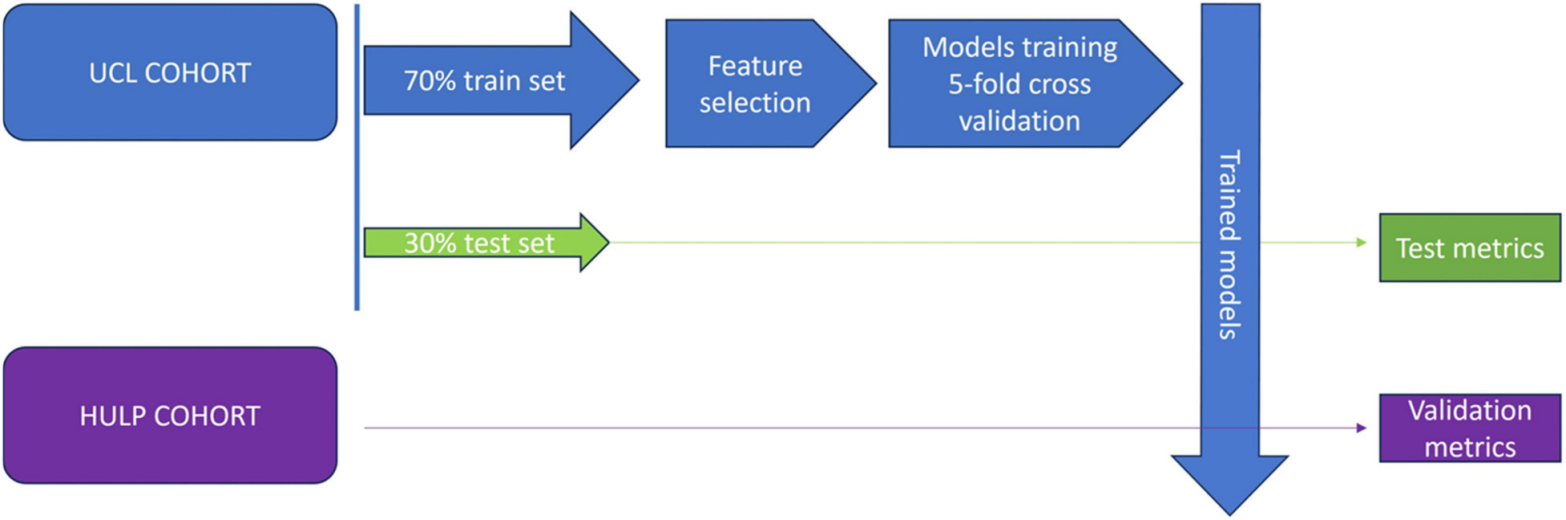
Machine learning scheme process.

Model development and validation procedures were conducted in parallel for both kPa defined fibrosis thresholds (≥7 KPa and ≥8 KPa), allowing for the evaluation and comparison of model performance using either cut-off. The machine learning scheme process is summarized in Figure 1.

The London cohort was randomly split into a training set (70%) and a test set (30%) using stratified sampling based on FIB-4 categories: <1.3, 1.3–2.67, and >2.67. This approach ensured proportional representation of each FIB-4 risk category in both the training and test subsets. The Madrid cohort was used exclusively for external validation.

Variables with more than 20% missing data in either of the two cohorts were excluded from the analysis. The percentage of missing data for each variable is presented in Supplementary Material. Missing values in the remaining variables were imputed using Python's iterative imputer, applying a Random Forest regressor for numerical variables and a Random Forest classifier for categorical variables.

Collinearity was assessed using a Variance Inflation Factor (VIF) > 10 and a correlation coefficient >0.7. Collinear variables were merged accordingly.

To minimise the risk of overfitting and potential bias introduced by the imputation process, several safeguards were implemented during model development. Variables with more than 20% missing data were excluded from the analysis, model development incorporated grid search with 5-fold cross-validation, model performance was evaluated on a held-out test dataset, and final model assessment was conducted using an independent external validation cohort.

Feature selection was performed using an Elastic Net logistic regression model with hyperparameter tuning via GridSearch. Model coefficients guided the selection of variables, and ten variables were selected for each model (KPA7 and KPA8) based on investigator judgment. The corresponding coefficients are presented in Supplementary Material.

### Machine learning algorithms

Model development included hyperparameter tuning via grid search combined with 5-fold cross-validation. The following algorithms were evaluated: Elastic Net, Support Vector Machine (SVM), Random Forest, Extreme Gradient Boosting (XGBoost), Neural Network (NN), and a stacking ensemble combining Elastic Net, SVM, XGBoost, and MLP Classifier (Ensemble). Grid search ranges and final selected parameters are detailed in Supplementary Material.

Machine learning models were developed and evaluated specifically in participants with FIB-4 scores between 1.3 and 2.67, corresponding to the diagnostic grey zone where additional risk stratification is clinically relevant.

### Performance metrics

For each model, the following performance metrics were calculated: accuracy, area under the receiver operating characteristic curve (AUC), recall or sensitivity, precision, F1-score, specificity, negative predictive value (NPV), and the confusion matrix. AUC values were not calculated for the clinical scores (e.g., FIB-4, APRI), as these are not predictive models and do not generate probability estimates. A significance level of 0.05 was used for all statistical analyses. Continuous variables were assessed for distribution, and as most variables were not normally distributed, results are presented as medians and interquartile ranges. Non-parametric tests were applied for bivariate analyses, including the Kruskal–Wallis test for continuous variables and the Chi-square or Fisher's exact test for categorical variables, as appropriate. All analyses were performed using Python version 3.11.5. No formal sample size calculation was performed, as this study was based on retrospective data available from the participating centres and should be considered exploratory.

## Results

### Population characteristics

The London cohort consisted of 229 participants. Demographic and laboratory characteristics are detailed in Table 1 and fibrosis scores are shown in Table 2. The median age was 52 (Interquartile range -IQR- 46–59) years, and participants were predominantly male (87.3%). All had undetectable HIV-RNA in plasma. The median TE measurement was 5.1 kPa (IQR 4.1–6.3), with 83.4% of participants having values below 7 kPa. The median CAP was 257 dB/m (IQR 215.0–299.0), being higher in the group with TE kPa ≥7, although this difference was not statistically significant. Regarding FIB-4 scores, 135 participants (59%) had values below 1.3, 79 (34.5%) between 1.3 and 2.67, and 15 (6.6%) above 2.67. Participants with TE ≥7 kPa had significantly higher FIB-4 scores compared to those with TE kPa <7. Liver biochemistry parameters (GGT, ALP, ALT, and AST) were also significantly elevated in the kPa ≥7 group (Table 1). There were no statistically significant differences between groups in metabolic parameters including total cholesterol, HDL, LDL, and triglycerides, although total cholesterol tended to be higher in the TE kPa <7 group and triglycerides higher in the TE kPa ≥7 group. All participants had undetectable HIV viral load at the time of analysis. Participants had been living with HIV for a median of 157 months (IQR 81.3–227.7) months, but it was longer in the TE kPa >7 group (*p* < 0.05). Diabetes mellitus was present in 16% of participants, and 3% had a history of HCV infection, with no significant differences observed between groups. However, significant differences were observed regarding excessive alcohol consumption (44% in TE kPa ≥7 vs. 29.8% in TE kPa <7) and BMI, which was slightly higher in the group with higher kPa.

**Table 1:** Participant characteristics.

Variable	London cohort				Madrid cohort			
	Missing	Overall	KPA≥7	KPA<7	Missing	Overall	KPA≥7	KPA<7
*n*	–	229	38	191	–	188	36	152
Age (years), median [IQR]	0	52.0 [46.0–59.0]	56.0 [50.2–61.0]*	51.0 [45.0–58.0]*	0	49.2 [41.9–54.2]	52.9 [43.9–57.2]	48.8 [41.6–53.9]
Gender (male), *n* (%) – Male	–	200 (87.3)	35 (92.1)	165 (86.4)	–	169 (89.9)	35 (97.2)	134 (88.2)
GGT (U/L), median [IQR]	36	58.0 [34.0–109.0]	89.5 [66.8–248.0]*	51.0 [31.0–93.0]*	2	51.5 [31.0–93.5]	50.0 [34.5–128.0]	52.0 [30.5–87.0]
ALP(U/L), median [IQR]	1	85.0 [68.8–103.5]	98.0 [79.0–116.0]*	83.0 [68.0–99.5]*	1	84.0 [66.0–99.5]	78.5 [65.8–92.0]	84.0 [67.0–100.5]
AST(U/L), median [IQR]	0	40.0 [31.0–51.0]	47.5 [37.0–80.5]*	38.0 [30.0–49.0]*	0	34.0 [27.0–43.0]	45.5 [34.8–56.2]*	32.5 [25.8–40.2]*
ALT(U/L), median [IQR]	0	59.0 [44.0–80.0]	77.0 [51.2–92.2]*	58.0 [44.0–77.5]*	0	48.5 [37.0–68.0]	66.0 [50.0–94.8]*	46.0 [35.0–62.2]*
Total bilirubin (mg/dL), median [IQR]	0	0.4 [0.3–0.6]	0.5 [0.3–0.7]	0.4 [0.3–0.6]	2	0.6 [0.5–0.7]	0.5 [0.4–0.6]	0.6 [0.5–0.7]
Albumin(g/dL), median [IQR]	0	4.7 [4.5–4.9]	4.7 [4.5–4.9]	4.8 [4.6–4.9]	46	4.4 [4.3–4.6]	4.5 [4.3–4.6]	4.4 [4.2–4.6]
Total cholesterol (mg/dL), median [IQR]	27	185.6 [159.5–215.6]	179.8 [161.4–212.7]	185.6 [159.5–216.6]	0	184.0 [161.8–203.2]	170.5 [156.0–185.5]*	189.5 [163.8–204.5]*
HDL cholesterol (mg/dL), median [IQR]	27	46.4 [38.7–58.0]	42.5 [37.7–58.0]	46.4 [38.7–57.0]	0	40.0 [34.0–49.0]	38.5 [33.8–44.5]	41.0 [34.0–50.0]
LDL cholesterol (mg/dL), median [IQR]	33	100.5 [81.2–127.6]	98.6 [82.2–122.8]	102.5 [81.2–127.6]	1	111.0 [92.0–128.5]	95.5 [81.0–122.2]*	113.0 [95.0–129.0]*
HDL ratio, median [IQR]	27	4.0 [3.1–4.9]	4.0 [3.4–5.0]	4.0 [3.1–4.9]	0	4.5 [3.7–5.3]	4.6 [3.7–5.2]	4.5 [3.7–5.3]
Triglycerides (mg/dL) median [IQR]	27	141.7 [97.4–221.4]	181.6 [121.8–290.1]*	141.7 [97.4–221.4]*	0	146.5 [97.0–213.8]	159.0 [109.0–234.0]	140.5 [97.0–205.0]
Platelets(×10⁹/L), median [IQR]	0	231.0 [198.0–269.0]	224.5 [192.2–273.8]	233.0 [199.0–269.0]	0	224.0 [189.0–264.5]	222.0 [188.5–264.5]	226.0 [189.8–264.5]
MCV(×10⁹/L), median [IQR]	0	94.2 [90.0–98.2]	96.3 [92.1–99.4]*	93.4 [89.8–97.6]*	5	94.0 [91.2–97.4]	93.7 [89.9–96.9]	94.1 [91.5–97.5]
HIV viral load (<50 copies/mL), %	0	100%	100%	100%	1	100%	100%	100%
HIV time since diagnosis(months), median [IQR]	6	157.2 [81.3–227.7]	194.1 [115.3–277.6]*	151.0 [80.5–213.6]*	0	150.4 [81.4–247.0]	144.7 [87.9–256.1]	150.6 [78.9–239.0]
Diabetes mellitus, *n* (%)	–	37 (16.2)	8 (21.1)	29 (15.2)	–	64 (34.0)	24 (66.7)*	40 (26.3)*
HCV infection, *n* (%)	–	7 (3.1)	1 (2.6)	6 (3.1)	–	0 (0.0)	0 (0.0)	0 (0.0)
Alcohol consumption, *n* (%)	–	74 (32.3)	17 (44.7)*	57 (29.8)*	–	16 (8.5)	5 (13.9)	11 (7.2)
BMI(kg/m^2^), median [IQR]	30	27.5 [24.8–30.7]	28.7 [26.8–33.3]*	27.1 [24.6–30.1]*	1	27.3 [24.3–29.7]	28.3 [25.4–31.4]*	26.4 [24.2–29.3]*

GGT, gamma-glutamyl transferase; ALP, alkaline phosphatase; AST, aspartate aminotransferase; ALT, alanine aminotransferase; BMI, body mass index; HDL, high-density lipoprotein; LDL, low-density lipoprotein; MCV, mean corpuscular volume; HIV, human immunodeficiency virus; HCV, hepatitis C virus; IQR, interquartile range.

*indicate statistically significant differences between the pair of values (*p* < 0.05, non-parametric tests were applied).

**Table 2:** Transient elastography and liver fibrosis markers .

Variable	London cohort				Madrid cohort			
	Missing	Overall	KPA≥7	KPA<7	Missing	Overall	KPA≥7	KPA<7
*n*	–	229	38	191	–	188	36	152
Liver stiffness; kPa, median [IQR]	0	5.1 [4.1–6.3]	8.9 [7.7–12.5]*	4.7 [4.0–5.8]*	0	5.3 [4.1–6.5]	8.9 [7.9–10.3]*	4.8 [4.0–5.8]*
CAP score; dB/m, median [IQR]	0	257.0 [215.0–299.0]	270.0 [216.2–330.5]	253.0 [215.5–295.0]	26	280.0 [233.0–323.0]	303.5 [258.0–353.0]*	271.0 [224.0–316.0]*
Fibrosis category, *n* (%)	–	–	–	–	–	–	–	–
<7	–	191 (83.4)	0 (0.0)	191 (100.0)	–	152 (80.9)	0 (0.0)	152 (100.0)
>7–<8.7	–	19 (8.3)	19 (55.3)	0 (0.0)	–	13 (6.9)	13 (36.1)	0 (0.0)
>8.7–<10.3	–	1 (0.4)	1 (2.6)	0 (0.0)	–	13 (6.9)	13 (36.1)	0 (0.0)
>10.3	–	18 (7.9)	18 (47.4)	0 (0.0)	–	10 (5.3)	10 (27.8)	0 (0.0)
FIB-4, median [IQR]	0	1.1 [0.9–1.7]	1.7 [0.9–1.9]*	1.1 [0.9–1.5]*	0	1.0 [0.8–1.4]	1.1 [0.9–1.7]	1.0 [0.7–1.3]
FIB-4 group, *n* (%)	–	–	–	–	–	–	–	–
<1.3	–	135 (59.0)	15 (39.5)*	120 (62.8)*	–	135 (71.8)	21 (58.3)*	114 (75.0)*
>1.3–<2.67	–	79 (34.5)	18 (47.4)*	61 (31.9)*	–	50 (26.6)	14 (38.9)*	36 (23.7)*
>2.67	–	15 (6.6)	5 (13.2)*	10 (5.2)*	–	3 (1.6)	1 (2.8)	2 (1.3)

KPA, kilopascal; CAP, controlled attenuation parameter; FIB-4, fibrosis-4 score; IQR, interquartile range.

*indicate statistically significant differences between the pair of values (*p* < 0.05, non-parametric tests were applied).

The Madrid cohort comprised 188 participants. Relevant characteristics are detailed in Table 1 and fibrosis scores are shown in Table 2. Participants had a median age of 49 (IQR 41.9–54.2) years and were predominantly male (90%). The median TE measurement was 5.3 kPa (IQR 4.1–6.5), with 80.9% having values below 7 kPa. The median CAP was 280 dB/m (IQR 233–323), significantly higher in the group with kPa ≥7. Median FIB-4 scores were significantly higher in the TE kPa ≥7 group, and liver biochemistry (AST and ALT) were significantly higher in this group as well. Regarding lipid parameters, participants with TE kPa <7 had higher levels of total cholesterol and LDL. All participants had undetectable HIV viral load (<50 copies/mL). The median known time since HIV diagnosis was 150 months (IQR 81.4–247.0). Diabetes was reported in 34% of participants, significantly higher (66%) in the TE kPa ≥7 group compared to the TE kPa <7 group (24%). None of the participants had a history of hepatitis C. Harmful alcohol consumption was observed in 8.5% of participants, being higher (14% vs. 7%) in the TE kPa ≥7 group, although this difference was not statistically significant. BMI was statistically significantly higher in the TE kPa ≥7 group.

When using 8 kPa as threshold for liver stiffness suggestive of fibrosis we observed similar results, as detailed in the Supplementary Material.

### Performance of non-invasive approaches

For the models designed to detect fibrosis at a cut-off of ≥7 kPa in individuals with a FIB-4 score >1.3, the variables selected using elastic net regression were GGT, BMI, alcohol consumption, triglycerides, age, ALP, time since HIV diagnosis, ALT, gender, and APRI score.

For the models designed to detect fibrosis at a cut-off of ≥8 kPa in individuals with a FIB-4 score >1.3, the variables selected were BMI, ALP, AST, GGT, alcohol consumption, total cholesterol, HDL ratio, triglycerides, age, and time since HIV diagnosis.

Table 3 summarizes the results of clinical scores and predictive models. In the context of fibrosis screening, sensitivity and negative predictive value are particularly important, as the primary goal is to identify individuals unlikely to have significant fibrosis. Clinical scores demonstrated high sensitivity but relatively low overall accuracy, reflecting their fixed threshold-based nature and the low prevalence of fibrosis in this screening population.

**Table 3:** Performance metrics of all models at 7 kPa and 8 kPa cut-offs for liver stiffness (fibrosis).

Metric	FIB-4	Elastic Net	SVM	RF	XGB	NN	Ensemble	APRI
7 kPa cut-off								
Accuracy	0.21/0.28	0.6/0.7	0.52/0.74	0.65/0.72	0.6/0.74	0.69/0.72	0.47/0.72	0.34/0.46
AUC	–	0.62/0.67	0.56/0.69	0.57/0.68	0.58/0.69	0.56/0.69	0.55/0.69	–
Recall	1/1	0.6/0.5	0.6/0.28	0.4/0.28	0.2/0.28	0.4/0.21	0.4/0.5	1/0.92
Precision	0.21/0.28	0.3/0.63	0.25/0.57	0.28/0.5	0.16/0.57	0.33/0.5	0.18/0.5	0.25/0.33
F1 score	0.35/0.43	0.4/0.56	0.35/0.38	0.33/0.36	0.18/0.38	0.36/0.3	0.25/0.55	0.4/0.49
Specificity	0/0	0.61/0.88	0.5/0.91	0.72/0.88	0.72/0.91	0.77/0.91	0.5/0.80	0.16/0.27
NPV	0/0	0.84/0.82	0.81/0.76	0.81/0.76	0.76/0.76	0.82/0.75	0.75/0.80	1/0.9
8 kPa cut-off								
Accuracy	0.12/0.26	0.62/0.8	0.62/0.8	0.9/0.74	0.79/0.76	0.91/0.78	0.58/0.8	0.37/0.44
AUC	–	0.84/0.72	0.84/0.68	0.76/0.67	0.80/0.69	0.85/0.7	0.84/0.72	–
Recall	1/1	0.66/0.23	0.66/0.23	0.66/0	0.66/0.15	0.66/0.15	0.66/0.23	0.66/0.92
Precision	0.12/0.26	0.2/1	0.2/1	0.66/0	0.33/0.66	0.66/1	0.18/1	0.12/0.3
F1 score	0.22/0.41	0.30/0.37	0.3/0.37	0.66/0	0.44/0.25	0.66/0.26	0.28/0.37	0.21/0.46
Specificity	0/0	0.61/1	0.61/1	0.95/1	0.80/0.97	0.95/1	0.57/1	0.33/0.27
NPV	0/0	0.92/0.78	0.92/0.78	0.95/0.74	0.94/0.76	0.95/0.77	0.92/0.78	0.87/0.9

AUC, area under the receiver operating characteristic curve; RF, random forest; XGB, extreme gradient boosting; NN, neural network; SVM, support vector machine; NPV, negative predictive value; FIB-4, fibrosis-4 score; APRI, aspartate aminotransferase to platelet ratio index. Each cell shows two values. The first value corresponds to the London test cohort, and the second value corresponds to the external validation cohort from Madrid.

For identifying participants with TE kPa ≥7, model accuracy was modest in the London cohort test set but performed better in the external validation cohort, achieving approximately 70%–75% accuracy and an AUC between 67% and 69% across all models. However, discrimination between false positives and false negatives was limited, with F1-scores barely exceeding 50% in some models. Confusion matrices for all models are presented in Supplementary Tables S5, S6, providing detailed counts of true positives, true negatives, false positives, and false negatives. These matrices indicate a clear tendency towards negative predictions, correctly classifying the majority but showing a high rate of misclassification among true positives.

In identifying participants with TE kPa ≥8 and a FIB-4 score >1.3, the models demonstrated better accuracy, ranging from 75% to 80% in the external validation cohort, with AUC values around 70% across models. However, the London cohort test set contained only three participants with TE ≥8 kPa, requiring cautious interpretation. Similar limitations were observed regarding discrimination between false positives and negatives, with F1-scores below 50% in the external validation cohort. A comparable pattern of negative predictions was noted, accurately classifying most participants but inadequately capturing true positive cases.

Given the screening purpose of these assessments, high sensitivity (recall) is desirable. The FIB-4 threshold demonstrated 100% sensitivity, reflecting its role as a rule-out screening tool, although its overall classification performance was limited due to low specificity. Notably, the APRI score exhibited high sensitivity, approximately 90%, in both test and validation cohorts, and showed strong negative predictive values ranging from 87% to 100%. None of the models demonstrated sensitivity and NPV levels comparable to those of the APRI score.

## Discussion

Our study aimed to address the uncertainty in risk stratification among people living with HIV with intermediate FIB-4 scores; specifically, how likely are such patients to have clinically significant fibrosis. We tested whether adding other approaches, including APRI and exploratory machine learning models, could improve identification of fibrosis.

MASLD is currently the most prevalent cause of chronic liver disease among people living with HIV, excluding those with chronic hepatitis B or C infection (4, 5). Moreover, in both of our cohorts, the median CAP value exceeded the widely recommended threshold of 248 dB/m for the diagnosis of hepatic steatosis (8), consistent with MASLD being a frequent cause of liver disease in these populations. We used TE as the reference standard for fibrosis. Although biopsy remains the gold standard, TE is widely included in clinical guidelines when non-invasive scores are abnormal and is commonly used in routine clinical practice (9).

Clinical guidelines suggest using non-invasive scores primarily to rule out liver fibrosis in at-risk populations such as people living with HIV or individuals with type 2 diabetes (9). In this screening context, sensitivity and negative predictive value are critical. Other diagnostic metrics, such as the area under the curve, are useful for evaluating overall performance, but they can be affected by sample imbalance and may obscure high false negative or false positive rates.

A range of machine learning models for liver fibrosis prediction have been developed in various clinical settings including general population screening (15), hepatitis C (16), and MASLD (17–19) or even for all causes of advanced cirrhosis (20). These previous models did not include people living with HIV, and some explicitly excluded them, and generally yielded promising results. For example, a Danish primary care study showed that models achieved over 90% sensitivity and NPV for identifying TE ≥8 kPa, even when validated against biopsy-confirmed F ≥ 2 cases. In the same study, APRI and FIB-4 showed sensitivity/NPV of 71%/91% and 49%/90% respectively for TE ≥8 kPa; and APRI retained 92% sensitivity and 80% NPV in the biopsy-confirmed subgroup (15). Another study comparing machine learning models and clinical scores in MASLD-related fibrosis (biopsy-confirmed) found comparable sensitivity and NPV: 72%/85% for the ML model, 87%/82% for FIB-4, and 38%/80% for APRI (17). A third study using ML for biopsy-defined fibrosis showed >90% sensitivity and NPV, similar to FIB-4, APRI, and TE—although the ML models in that case included TE as a predictive variable (19).

To our knowledge, this is the first attempt to apply exploratory machine learning models specifically for hepatic fibrosis detection in people living with HIV and intermediate FIB-4 score. Their performance was limited compared with simple non-invasive scores. The models did not achieve the expected performance in terms of sensitivity and NPV, either for TE thresholds of ≥7 or ≥8 kPa. While some models demonstrated acceptable AUC and accuracy, this was likely due to a tendency toward negative classification. Most models predicted “no fibrosis” for nearly all patients, resulting in good general metrics but poor control of false positives and false negatives. This is especially problematic in a screening setting, where failing to identify true cases of fibrosis could have clinical consequences. One possible explanation is the complexity of distinguishing hepatic fibrosis in people living with HIV (1).

Several studies have explored the performance of non-invasive scores such as FIB-4 and APRI for liver fibrosis detection in people with HIV with MASLD. In one study using liver biopsy as the reference standard (F ≥ 2), both FIB-4 (cut-off 1.45) and APRI (cut-off 0.5) demonstrated good performance, with sensitivities and NPVs of 87%/89% for FIB-4% and 87%/92% for APRI. Correct classification was achieved in 61% and 73% of participants, respectively. Notably, these results outperformed TE, which showed a sensitivity of 80% and NPV of 75% (13). Another study evaluating liver fibrosis in in people with HIV and MASLD also found similar AUC values for FIB-4, APRI, and TE in detecting mild fibrosis (14).

Although our machine learning models were unable to effectively discriminate risk for fibrosis in those with intermediate FIB-4 score (i.e., 1.3–2.67), APRI demonstrated strong performance across both cohorts. Using a cut-off of >0.5, APRI achieved 100% sensitivity and NPV for TE kPA≥7 in the London cohort, and 90% in the external validation cohort. For TE kPA≥8, although the test sample was smaller, APRI still achieved 66% sensitivity and 87% NPV in the London cohort, and 90% sensitivity and NPV in the Madrid cohort. Applying APRI in a sequential manner, after a FIB-4 score >1.3, led to improved classification. This stepwise approach enhanced correct classification in 13%–18% of participants with TE kPa≥7, and 18%–25% for TE kPa≥8 across both cohorts. Such a strategy may offer a practical, low-cost improvement to fibrosis screening pathways, particularly in settings with limited access to imaging or biopsy. Our findings highlight that, in this context, routinely available scores such as APRI may offer greater practical value than more complex modelling approaches.

These findings are consistent with previous studies evaluating non-invasive fibrosis assessment in people living with HIV and other low-prevalence populations, where simpler scores such as APRI and FIB-4 have demonstrated robust rule-out performance. In such settings, more complex modelling approaches do not necessarily translate into improved clinical utility, particularly when the primary objective is to exclude significant fibrosis. Our results support the concept that, in real-world screening populations, emphasis should be placed on sensitivity and negative predictive value rather than overall classification accuracy, and that additional model complexity may offer limited incremental benefit over established clinical tools.

Our study has some limitations. The prevalence of advanced fibrosis in both cohorts was relatively low, reflecting the characteristics of a screening population. This resulted in class imbalance, which likely contributed to the tendency of machine learning models to favour negative predictions. In low-prevalence screening populations such as ours, this can lead to apparently acceptable overall performance metrics while masking poor sensitivity for detecting true fibrosis cases. In addition, the development cohort was relatively small for machine learning modelling, which may have limited the ability of the algorithms to capture complex relationships between variables. Calibration of the machine learning models was not formally assessed. Given the relatively small number of fibrosis cases in both cohorts, particularly for the ≥8 kPa threshold, calibration curves derived from probability binning would likely have been unstable and difficult to interpret. We are working on larger pooled cohorts with a wider range of liver disease to enhance model robustness. In addition, TE, although recommended in clinical practice, is not as reliable as liver biopsy and may misclassify patients. Nonetheless, it remains a key decision-making tool in an era when few patients undergo biopsy. Additionally, the validation set for the London cohort was relatively small, limiting assessment of generalisability. The difference in harmful alcohol consumption between the two cohorts was significant. Alcohol use was included in the models, as an important cause of liver disease, and a contributory factor in many people living with HIV (21, 22).

## Conclusion

This study addressed the diagnostic grey zone of FIB-4 in people living with HIV, where decisions about referral for further assessment such as elastography remain uncertain. Our findings show that while machine learning models offered only modest discrimination, the sequential application of APRI after FIB-4 provided a simple and pragmatic improvement in identifying individuals at risk of fibrosis. This low-cost strategy may enhance early detection and guide more efficient referral pathways among those living with HIV under routine care. Further research with larger prospective cohorts is needed to further refine non-invasive screening algorithms, including exploring integration of novel biomarkers.

## Data Availability

The raw data supporting the conclusions of this article will be made available by the authors, without undue reservation.
