# Supplementary material for: Optimising non-invasive screening for hepatic fibrosis in people living with HIV and intermediate FIB-4 scores
Source: Front Health Serv. 2026 Apr 29;6:1807690. doi: 10.3389/frhs.2026.1807690 (PMC13168209; doi:10.3389/frhs.2026.1807690)
Supplement: Supplementary file 1 [file Table1.docx]

## **Supplementary Material**

## **Optimising Non-Invasive Screening for Hepatic Fibrosis in People Living with HIV and Intermediate FIB-4 Scores.**

**Supplementary Section 1 – Data Completeness**

**Supplementary Table S1. Percentage of missing data for candidate variables in the London and Madrid cohorts**

|  | **% Missing values** | |
| --- | --- | --- |
|  | **London** | **Madrid** |
| **KPA** | 0.00 | 0.00 |
| **CAP** | 0.00 | 13.83 |
| **Age** | 0.00 | 0.00 |
| **Gender** | 0.00 | 0.00 |
| **Albumin** | **0.00** | **24.47** |
| **ALP** | 0.44 | 0.53 |
| **ALT** | 0.00 | 0.00 |
| **AST** | 0.00 | 0.00 |
| **GGT** | 15.72 | 1.06 |
| **Bilirubin** | 0.00 | 1.06 |
| **Total cholesterol** | 11.79 | 0.00 |
| **HDL cholesterol** | 11.79 | 0.00 |
| **HDL-cholesterol ratio** | 11.79 | 0.00 |
| **LDL cholesterol** | 14.41 | 0.53 |
| **Triglycerides** | 11.79 | 0.00 |
| **Creatinine** | 1.31 | 0.53 |
| **Urea** | **1.31** | **92.55** |
| **Hemoglobin** | **0.00** | **31.38** |
| **MVC** | 0.00 | 2.66 |
| **Platelet count** | 0.00 | 0.00 |
| **White cell count** | **0.00** | **33.51** |
| **Sodium** | **0.00** | **54.26** |
| **HIV viral load** | 2.62 | 0.53 |
| **HIV time** | 1.31 | 0.00 |
| **DM** | 1.31 | 0.00 |
| **VHC** | 0.00 | 0.00 |
| **BMI** | 13.10 | 0.53 |
| **Alcohol** | 14.41 | 4.26 |
| **FIB4** | 0.00 | 0.00 |
| **FIB4_group** | 0.00 | 0.00 |
| **APRI** | 0.00 | 0.00 |
| **APRI_group** | 0.00 | 0.00 |

KPA: kilopascals; CAP: controlled attenuation parameter; er; HepB Ag: hepatitis B surface antigen; HepB dna: hepatitis B viral load; HepB time: time since hepatitis B diagnosis; ALP: alkaline phosphatase; ALT: alanine aminotransferase; AST: aspartate aminotransferase; GGT: gamma-glutamyl transferase; HDL cholesterol: high-density lipoprotein cholesterol; LDL cholesterol: low-density lipoprotein cholesterol; MVC: mean corpuscular volume; HIV time: time since human immunodeficiency virus diagnosis; DM: diabetes mellitus; VHC: hepatitis C virus; BMI: body mass index; FIB4: fibrosis-4 index; FIB4_group: fibrosis-4 index category; APRI: aspartate aminotransferase to platelet ratio index; APRI_group: aspartate aminotransferase to platelet ratio index category.

**Supplementary Section 2 – Cohort Characteristics by Fibrosis Threshold**

**Supplementary Table S2. Baseline characteristics stratified by fibrosis status (TE cut-off ≥8 kPa)**

| **VARIABLES** | **LONDON COHORT** | | | | **MADRID COHORT** | | | |
| --- | --- | --- | --- | --- | --- | --- | --- | --- |
|  | **Missing** | **Overall** | **KPA <8** | **KPA ≥8** | **Missing** | **Overall** | **KPA <8** | **KPA ≥8** |
| N | – | 229 | 203 | 26 | – | 188 | 162 | 26 |
| Age (years), median  [IQR] | 0 | 52.0  [46.0–59.0] | 52.0  [45.0–59.0] | 53.5  [48.0–60.0] | 0 | 49.2  [41.9–54.2] | 48.7  [41.3–53.8] | 54.6  [50.5–58.6] |
| Gender, n (%) – Male | – | 200 (87.3) | 175 (86.2) | 25 (96.2) | – | 169 (89.9) | 143 (88.3) | 26 (100.0) |
| Gender, n (%) – Female | – | 29 (12.7) | 28 (13.8) | 1 (3.8) | – | 19 (10.1) | 19 (11.7) | 0 (0.0) |
| GGT(U/L), median  [IQR] | 36 | 58.0  [34.0–109.0] | 51.0  [33.0–94.5] | 111.0  [72.0–276.2] | 2 | 51.5  [31.0–93.5] | 50.5  [30.0–87.5] | 52.0  [41.0–129.0] |
| ALP(U/L), median  [IQR] | 1 | 85.0  [68.8–103.5] | 84.0  [68.0–100.5] | 108.0  [80.0–116.0] | 1 | 84.0  [66.0–99.5] | 84.0  [67.0–101.0] | 73.5  [64.2–88.5] |
| AST(U/L), median  [IQR] | 0 | 40.0  [31.0–51.0] | 38.0  [30.0–50.0] | 48.0  [41.2–92.0] | 0 | 34.0  [27.0–43.0] | 33.0  [26.2–41.0] | 45.5  [35.5–54.8] |
| Total bilirubin  (mg/dL), median  [IQR] | 0 | 0.4  [0.3–0.6] | 0.4  [0.3–0.6] | 0.5  [0.3–0.7] | 2 | 0.6  [0.5–0.7] | 0.6  [0.4–0.7] | 0.5  [0.5–0.8] |
| Albumin  (mg/dL), median  [IQR] | 0 | 4.7  [4.5–4.9] | 4.7  [4.5–4.9] | 4.7  [4.5–4.9] | 46 | 4.4  [4.3–4.6] | 4.5  [4.3–4.6] | 4.4  [4.3–4.6] |
| Total cholesterol  (mg/dL), median  [IQR] | 27 | 185.6  [159.5–215.6] | 185.6  [162.4–216.6] | 174.0  [154.7–205.0] | 0 | 184.0  [161.8–203.2] | 189.0  [163.0–206.8] | 170.5  [154.0–183.0] |
| HDL cholesterol  (mg/dL), median  [IQR] | 27 | 46.4  [38.7–58.0] | 46.4 [38.7–58.0] | 38.7  [34.8–50.3] | 0 | 40.0  [34.0–49.0] | 41.0 [35.0–49.0] | 37.0  [31.5–46.8] |
| LDL cholesterol  (mg/dL), median  [IQR] | 33 | 100.5  [81.2–127.6] | 100.5  [81.2–127.6] | 96.7  [79.3–125.7] | 1 | 111.0  [92.0–128.5] | 113.0  [95.0–129.0] | 92.0  [84.5–118.8] |
| HDL ratio, median  [IQR] | 27 | 4.0  [3.1–4.9] | 4.0  [3.1–4.9] | 4.3  [3.5–5.3] | 0 | 4.5  [3.7–5.3] | 4.5  [3.7–5.3] | 4.6  [3.5–5.2] |
| Triglycerides  (mg/dL), median  [IQR] | 27 | 141.7  [97.4–221.4] | 141.7  [97.4–221.4] | 186.0  [124.0–283.4] | 0 | 146.5  [97.0–213.8] | 144.0  [97.0–205.8] | 159.0  [112.2–231.0] |
| Platelets median  [IQR] | 0 | 231.0  [198.0–269.0] | 230.0  [198.0–269.0] | 245.5  [193.5–277.2] | 0 | 224.0  [189.0–264.5] | 226.5  [193.2–266.8] | 219.5  [187.0–259.8] |
| MCV (fL), median  [IQR] | 0 | 94.2  [90.0–98.2] | 93.7  [90.0–97.8] | 96.0 [91.3–98.8] | 5 | 94.0  [91.2–97.4] | 94.0  [91.4–97.4] | 94.0  [90.7–97.0] |
| HIV viral load (<50 copies/mL), % | 0 | 100% | 100% | 100% | 1 | 100% | 100% | 100% |
| HIV time since diagnosis (months), median  [IQR] | 6 | 157.2  [81.3–227.7] | 152.8  [80.2–214.0] | 204.7  [123.9–292.6] | 0 | 150.4  [81.4–247.0] | 141.9  [77.4–237.1] | 226.1  [119.3–265.5] |
| Diabetes mellitus, n (%) | – | 37 (16.2) | 30 (14.8) | 7 (26.9) | – | 64 (34.0) | 45 (27.8) | 19 (73.1) |
| HCV infection, n (%) | – | 7 (3.1) | 7 (3.4) | 0 (0.0) | – | 0 (0.0) | 0 (0.0) | 0 (0.0) |
| Alcohol consumption, n (%) | – | 74 (32.3) | 61 (30.0) | 13 (50.0) | – | 16 (8.5) | 12 (7.4) | 4 (15.4) |
| BMI  (kg/m²), median  [IQR] | 30 | 27.5  [24.8–30.7] | 27.2  [24.8–30.3] | 28.7  [26.2–32.5] | 1 | 27.3  [24.3–29.7] | 26.6  [24.1–29.3] | 29.3  [26.8–33.3] |

**Legend:** GGT: gamma-glutamyl transferase; ALP: alkaline phosphatase; AST: aspartate aminotransferase; BMI: body mass index; HDL: high-density lipoprotein; LDL: low-density lipoprotein; MCV: mean corpuscular volume; HIV: human immunodeficiency virus; HCV: hepatitis C virus; IQR: interquartile range.

**Supplementary Table S3. Transient elastography and fibrosis marker distribution (TE cut-off ≥8 kPa)**

| Variable | **London** | | | | **Madrid** | | | |
| --- | --- | --- | --- | --- | --- | --- | --- | --- |
|  | **Missing** | **Overall** | **KPA <8** | **KPA ≥8** | **Missing** | **Overall** | **KPA <8** | **KPA ≥8** |
| n | – | 229 | 203 | 26 | – | 188 | 162 | 26 |
| Liver stiffness  kPa, median  [IQR] | 0 | 5.1  [4.1–6.3] | 4.8  [4.1–6.1] | 11.2  [8.7–14.4] | 0 | 5.3  [4.1–6.5] | 4.8  [4.1–5.9] | 10.1  [8.8–11.6] |
| CAP score  dB/m, median  [IQR] | 0 | 257.0  [215.0–299.0] | 254.0  [215.5–296.5] | 270.0  [216.5–338.5] | 26 | 280.0  [233.0–323.0] | 271.0  [224.5–316.0] | 315.5  [281.0–353.0] |
| Fibrosis category, n (%) | – | – | – | – | – | – | – | – |
| Category 1 | – | 191 (83.4) | 191 (94.1) | 0 (0.0) | – | 152 (80.9) | 152 (93.8) | 0 (0.0) |
| Category 2 | – | 21 (9.2) | 12 (5.9) | 9 (34.6) | – | 21 (11.2) | 10 (6.2) | 11 (42.3) |
| Category 3 | – | 7 (3.1) | 0 (0.0) | 7 (26.9) | – | 11 (5.9) | 0 (0.0) | 11 (42.3) |
| Category 4 | – | 10 (4.4) | 0 (0.0) | 10 (38.5) | – | 4 (2.1) | 0 (0.0) | 4 (15.4) |
| FIB-4, median  [IQR] | 0 | 1.1  [0.9–1.7] | 1.1  [0.9–1.6] | 1.8  [0.9–1.9] | 0 | 1.0  [0.8–1.4] | 0.9  [0.7–1.3] | 1.4  [1.0–1.9] |
| FIB-4 group, n (%) | – | – | – | – | – | – | – | – |
| <1.3 | 1 | 135 (59.0) | 123 (60.6) | 12 (46.2) | 1 | 135 (71.8) | 123 (75.9) | 12 (46.2) |
| 1.3–2.67 | 2 | 79 (34.5) | 70 (34.5) | 9 (34.6) | 2 | 50 (26.6) | 37 (22.8) | 13 (50.0) |
| >2.67 | 3 | 15 (6.6) | 10 (4.9) | 5 (19.2) | 3 | 3 (1.6) | 2 (1.2) | 1 (3.8) |

**Legend:** KPA: kilopascal; CAP: controlled attenuation parameter; FIB-4: fibrosis-4 score; IQR: interquartile range

**Supplementary Section 3 – Feature Selection and Model Inputs**

**Multicollinearity assessment.** Multicollinearity among candidate variables was assessed using both the Variance Inflation Factor (VIF) and Pearson correlation coefficients. Variables with VIF >10 included the constant term, AST, total cholesterol, LDL cholesterol, FIB-4, and APRI. Additionally, several variable pairs showed strong correlations (r >0.7), including AST with FIB-4, AST with APRI, total cholesterol with LDL cholesterol, HDL ratio with HDL cholesterol, and FIB-4 with APRI.

**Supplementary Figure S1. Feature coefficients from Elastic Net model predicting TE ≥7 kPa.** Top features selected by the Elastic Net logistic regression model for the ≥7 kPa outcome. The model was optimised using cross-validation (CV score = 0.685). Hyperparameters: C = 1.0, l1_ratio = 0.9.


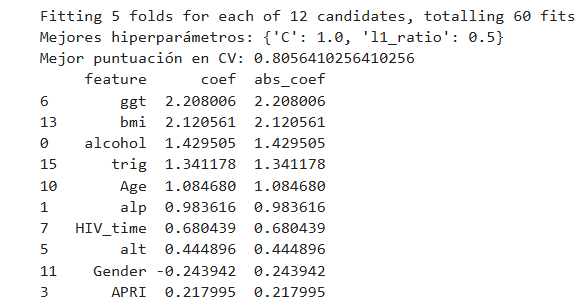


**Supplementary Figure S2. Feature coefficients from Elastic Net model predicting TE ≥8 kPa** Top features selected by the Elastic Net logistic regression model for the ≥8 kPa outcome. The model was optimised using cross-validation (CV score = 0.70). Hyperparameters: C = 1.0, l1_ratio = 0.9.


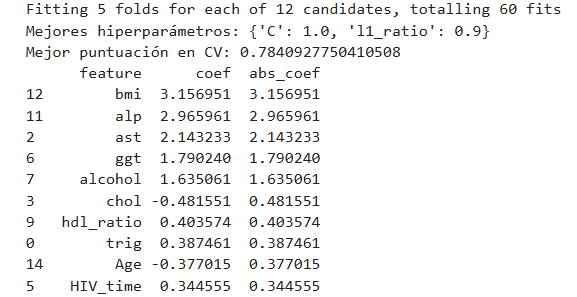


**Supplementary Section 4 – Non-invasive Score Calculation**

**Calculation of Non-Invasive Fibrosis Scores.** The non-invasive fibrosis scores used in this study were calculated as follows:

APRI = (AST / ULN) / platelet count × 100

FIB-4 = (Age × AST) / (Platelet count × √ALT)

**Supplementary Section 5 – Model Optimisation**

**Supplementary Table S4. Hyperparameter grid and optimal parameters for machine learning models predicting TE ≥7 kPa**

| Model | Grid Parameters | Best Parameters |
| --- | --- | --- |
| Elastic Net | 'C': [0.001, 0.01, 0.1, 1.0, 10, 100]; 'l1_ratio': [0.1, 0.5, 0.7, 0.9] | {'C': 10, 'l1_ratio': 0.9} |
| SVM | 'C': [0.001, 0.01, 0.1, 1, 10, 100, 1000]; 'kernel': ['linear', 'rbf']; 'gamma': ['scale', 'auto', 0.001, 0.01, 0.1, 1, 10] | {'C': 1000, 'gamma': 0.01, 'kernel': 'rbf'} |
| Random Forest | 'n_estimators': [50, 100, 200, 500]; 'max_depth': [5, 10, 20]; 'min_samples_split': [2, 5, 10]; 'min_samples_leaf': [1, 2, 4]; 'class_weight': ['balanced']; 'max_features': ['sqrt', 'log2']; 'bootstrap': [True, False] | {'bootstrap': True, 'class_weight': 'balanced', 'max_depth': 10, 'max_features': 'sqrt', 'min_samples_leaf': 4, 'min_samples_split': 10, 'n_estimators': 100} |
| XGBoost | 'n_estimators': [50, 100, 200]; 'max_depth': [3, 5, 7]; 'learning_rate': [0.001, 0.01, 0.1, 0.2]; 'subsample': [0.6, 0.8, 1.0]; 'colsample_bytree': [0.6, 0.8, 1.0]; 'scale_pos_weight': [imbalance_ratio] | {'colsample_bytree': 0.6, 'learning_rate': 0.2, 'max_depth': 7, 'n_estimators': 100, 'scale_pos_weight': 5.07, 'subsample': 0.6} |
| MLP | 'hidden_layer_sizes': [(32,), (64,), (32,32), (64,32)]; 'alpha': [0.0001, 0.001, 0.01]; 'learning_rate_init': [0.001, 0.01]; 'activation': ['relu', 'tanh']; 'solver': ['adam'] | {'activation': 'relu', 'alpha': 0.0001, 'hidden_layer_sizes': (32,), 'learning_rate_init': 0.001, 'solver': 'adam'} |

**Supplementary Section 6 – Model Performance**

**Supplementary Table S5. Confusion matrices for models predicting liver stiffness ≥7 kPa**

| **Model** | **Dataset** | **True Negative (TN)** | **False Positive (FP)** | **False Negative (FN)** | **True Positive (TP)** |
| --- | --- | --- | --- | --- | --- |
| Elastic Net | Test (London) | 11 | 7 | 2 | 3 |
| Elastic Net | Validation (Madrid) | 32 | 4 | 7 | 7 |
| SVM | Test (London) | 9 | 9 | 2 | 3 |
| SVM | Validation (Madrid) | 33 | 3 | 10 | 4 |
| MLP | Test (London) | 9 | 9 | 2 | 3 |
| MLP | Validation (Madrid) | 30 | 6 | 8 | 6 |
| Stacking | Test (London) | 11 | 7 | 3 | 2 |
| Stacking | Validation (Madrid) | 32 | 4 | 10 | 4 |
| Random Forest | Test (London) | 10 | 8 | 3 | 2 |
| Random Forest | Validation (Madrid) | 34 | 2 | 11 | 3 |
| XGBoost | Test (London) | 10 | 8 | 3 | 2 |
| XGBoost | Validation (Madrid) | 34 | 2 | 10 | 4 |
| APRI | Test (London) | 13 | 5 | 3 | 2 |
| APRI | Validation (Madrid) | 31 | 5 | 6 | 8 |
| FIB-4 | Test (London) | 1 | 17 | 0 | 5 |
| FIB-4 | Validation (Madrid) | 0 | 36 | 0 | 14 |

**Legend:** TN: true negatives; FP: false positives; FN: false negatives; TP: true positives

**Supplementary Table S6. Confusion matrices for models predicting liver stiffness ≥8 kPa.** Confusion matrices for all evaluated models in the test dataset (London cohort) and external validation dataset (Madrid cohort). Models were evaluated in participants with FIB-4 scores between 1.3 and 2.67, representing the diagnostic grey zone where additional risk stratification is clinically relevant.

| **Model** | **Dataset** | **True Negative (TN)** | **False Positive (FP)** | **False Negative (FN)** | **True Positive (TP)** |
| --- | --- | --- | --- | --- | --- |
| Elastic Net | Test (London) | 15 | 5 | 0 | 3 |
| Elastic Net | Validation (Madrid) | 39 | 5 | 5 | 1 |
| SVM | Test (London) | 14 | 6 | 0 | 3 |
| SVM | Validation (Madrid) | 39 | 5 | 6 | 0 |
| MLP | Test (London) | 13 | 7 | 0 | 3 |
| MLP | Validation (Madrid) | 36 | 8 | 4 | 2 |
| Stacking | Test (London) | 15 | 5 | 2 | 1 |
| Stacking | Validation (Madrid) | 38 | 6 | 5 | 1 |
| Random Forest | Test (London) | 15 | 5 | 1 | 2 |
| Random Forest | Validation (Madrid) | 39 | 5 | 5 | 1 |
| XGBoost | Test (London) | 15 | 5 | 1 | 2 |
| XGBoost | Validation (Madrid) | 40 | 4 | 5 | 1 |
| APRI | Test (London) | 15 | 5 | 0 | 3 |
| APRI | Validation (Madrid) | 39 | 5 | 2 | 4 |
| FIB-4 | Test (London) | 1 | 19 | 0 | 3 |
| FIB-4 | Validation (Madrid) | 0 | 44 | 0 | 6 |

**Legend:** TN: true negatives; FP: false positives; FN: false negatives; TP: true positives. Test dataset corresponds to the London cohort and validation dataset corresponds to the Madrid cohort.
